# Role of Optimization in RNA–Protein-Binding Prediction

**DOI:** 10.3390/cimb46020087

**Published:** 2024-02-04

**Authors:** Shrooq Alsenan, Isra Al-Turaiki, Mashael Aldayel, Mohamed Tounsi

**Affiliations:** 1 Information Systems Department, College of Computer and Information Sciences, Princess Nourah bint Abdulrahman University, P.O. Box 84428, Riyadh 11671, Saudi Arabia; 2 Department of Computer Science, College of Computer and Information Sciences, King Saud University, Riyadh 11653, Saudi Arabia; ialturaiki@ksu.edu.sa; 3 Information Technology Department, College of Computer and Information Sciences, King Saud University, Riyadh 11451, Saudi Arabia; maldayel@ksu.edu.sa; 4 Department of Computer Science, College of Computer and information Sciences, Prince Sultan University, P.O. Box 66833, Riyadh 12435, Saudi Arabia; mtounsi@psu.edu.sa

**Keywords:** RNA-binding proteins, bioinformatics, proteins, deep learning, convolutional neural network (CNN), optimization, grid search, random search optimizer, Bayesian optimizer, machine learning, artificial intelligence

## Abstract

RNA-binding proteins (RBPs) play an important role in regulating biological processes, such as gene regulation. Understanding their behaviors, for example, their binding site, can be helpful in understanding RBP-related diseases. Studies have focused on predicting RNA binding by means of machine learning algorithms including deep convolutional neural network models. One of the integral parts of modeling deep learning is achieving optimal hyperparameter tuning and minimizing a loss function using optimization algorithms. In this paper, we investigate the role of optimization in the RBP classification problem using the CLIP-Seq 21 dataset. Three optimization methods are employed on the RNA–protein binding CNN prediction model; namely, grid search, random search, and Bayesian optimizer. The empirical results show an AUC of 94.42%, 93.78%, 93.23% and 92.68% on the ELAVL1C, ELAVL1B, ELAVL1A, and HNRNPC datasets, respectively, and a mean AUC of 85.30 on 24 datasets. This paper’s findings provide evidence on the role of optimizers in improving the performance of RNA–protein binding prediction.

## 1. Introduction

RNA-binding proteins (RBPs) are proteins that bind to the double- or single-stranded RNA in cells and participate in forming ribonucleoprotein complexes. It is estimated that there are more than 1500 RNA-binding proteins in the human genome [1]. The interaction of proteins and RNA is essential for regulating gene expression at transcriptional and post-transcriptional levels [2]. Dysregulation in the interaction can cause cellular defects, leading to many diseases, such as cancerous tumors [3], genetic diseases [4], and neurological disorders, such as Alzheimer’s disease [5]. Thus, it is important to identify RNA–protein binding sites in order to understand the binding effect.

Various experimental techniques are available for the detection of RBP sites, such as crosslinking immunoprecipitation (HITS-CLIP), light-activated-ribonucleotide-enhanced crosslinking and immunoprecipitation (PAR-CLIP), and individual-nucleotide resolution crosslinking and immunoprecipitation (iCLIP) [6].

Although effective, such high-throughput technologies are costly, time-consuming, and sensitive to experimental variance. In addition to high-throughput sequencing methods that rely on crosslinking and immunoprecipitation, other experimental techniques are also used, such as electrophoretic mobility shift assays (EMSAs) [7], high-throughput imaging [8], and RNA-Bind-n-Seq [9].

Some studies propose enhancements to the scientific protocol in RBP binding sites. For example, the detailed steps of the PAR-CLIP protocol presented by Garzia et al. [10] to enhance and improve the experimental steps. For the enrichment of RBP binding sites, the study suggests using the kernel density algorithm PARalyzer to detect the density of thymidine-to-cytidine conversions that help in discriminating crosslinked from co-isolated non-crosslinked input RNAs. However, optimization algorithms for RNA–protein binding sites prediction were not employed.

Recently, several computational algorithms have been introduced in order to enhance the accuracy of RBP-binding-site detection. Deep learning has recently been a major research field in solving computational biology problems [11,12]. This is due to its capacity to uncover hidden patterns in complex biological data [13]. In particular, convolutional neural networks (CNNs) have demonstrated promising results for bioinformatics prediction tasks, including peptides [14], splice sites [15], and RNA–protein binding sites [6]. CNNs have been the primary mechanism for extracting RBP information in deep-learning-based approaches [6].

For example, iDeep trains a hybrid deep network with deep belief networks (DBNs) and CNNs using the CLIP-Seq datasets [16]. iDeepE combines a global and local CNNs to predict RNA–protein binding sites and motifs using only RNA sequences. The research investigated the impact of parameter optimization on the performance of the RNA–protein binding model using grid search, which improved the dropout probability, window size, and regularization [17]. DFpin is a cascade structure of deep forest learning for protein-binding-site prediction with feature-based redundancy removal [18]. The method works by analyzing the mono-nucleotide composition of the RNA fragments. DeepBtoD is ensemble learning for RNA-binding protein prediction using integrated deep learning [19]. This deep learning method learns high-level features using a self-attention mechanism and integrates local and global information from RNA sequences to enhance the prediction.

The advent of high-throughput sequencing technology has yielded vast datasets. The incorporation of deep learning algorithms in this field presents an opportunity to generate entirely data-driven predictions of binding sites. While statistical methods can be effectively utilized to identify enriched peaks in crosslinking and immunoprecipitation experiments, the application of CNNs provides distinct advantages. CNNs are capable of automatically learning features directly from raw data. This is particularly useful in situations where the relationships between genomic regions and binding events are not easily characterized by manually crafted features. Additionally, CNNs excel in capturing intricate spatial patterns within the data.

CNNs are a type of deep neural network with an architecture of many convolutions, pooling, and fully connected layers. There are numerous parameters to be set in order to control the model learning process. Those parameters are referred to as *hyperparameters*, and include the number of hidden layers, activation function, learning rate, batch size, and optimizer. The performance of CNNs is highly dependent on setting the optimal values for the network hyperparameters. With the increasing complexity of data, this task is far from trivial. Hyperparameter optimization can be achieved using various methods, such as manual setting, grid search, random search, Bayesian optimizer [20], and Tree Parzen Estimators [21].

Random search is a simple algorithm and easy to implement. Its basic idea is testing random inputs of the objective function. Its efficacy stems from its lack of reliance on prior assumptions about the underlying structure of the problem, as opposed to methods like Bayesian optimization. Bayesian optimizer is a sequential model-based approach that aims to identify the global optimum with the fewest trials possible. In Bayesian optimizer, a prior assumption about the potential objective functions is established. It then undergoes successive improvement using the Bayesian posterior. Bayesian optimizer has demonstrated success in many applications such as environmental monitoring, information extraction, and experimental design [22].

Several studies have demonstrated the effectiveness of optimization and heuristic methods such as Bayesian optimizer, grid search, and random search methods in improving model prediction. Calvet et al. [23] demonstrated the effect of optimization algorithms in solving bioinformatics problems such as molecular docking and protein structure prediction. They suggested the combination of multiple heuristic and optimization algorithms to solve modern computational problems. In classifying bioactive compounds, Czarnecki et al. [24] demonstrated that random search optimization can lead to improved performance compared to grid and heuristic approaches. Bayesian optimizer has attracted interest due to its usefulness in tuning hyperparameters for deep learning [25]. It is used to fine-tune the search where domain knowledge, such as parameter selection, is complemented with computational approaches and model analysis. It is an iterative global optimization method which aims to maximize an objective function over a limited set of variables and constraints [26].

Bayesian optimizer had been applied in biochemical applications, for the production of mRNA molecules [26], and in bioinformatics, for the assembly of RNA sequences [25] and for improving genetic expression quantitative trait loci (eQTL) analysis [27], aptamer (RNA/DNA oligonucleotide molecules) discovery [28], and generating RNA secondary structures [29].

**Figure 1:**
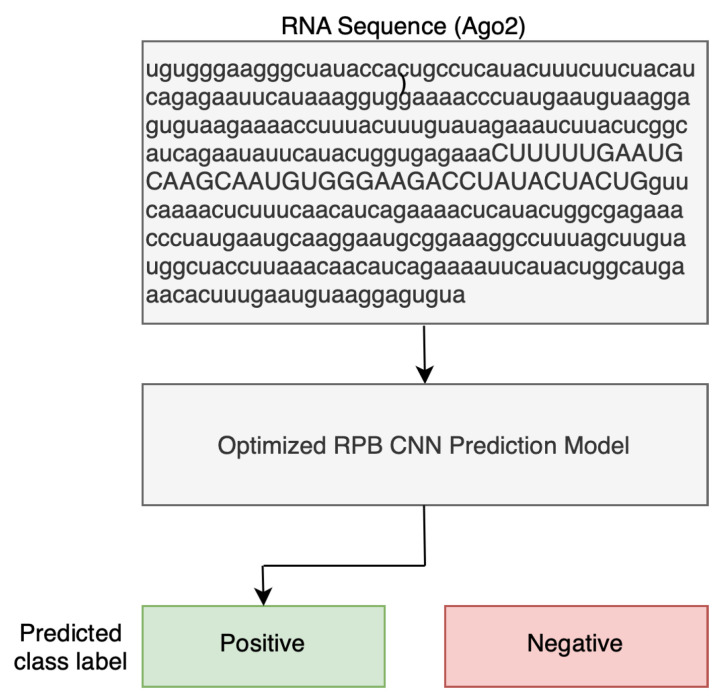
RBP binding sites as a binary classification problem.

Many solutions based on CNNs for determining RBP sites have been introduced in the literature [1,6,13,30]. The task of RBP-binding-site detection is a binary classification problem, where it is required to classify input sequences into either *positive* (binding) or *negative* (non-binding), as shown in Figure 1. Given an RNA sequence, our model can take the RNA sequence (input features) as an input representing the genetic information stored in RNA, and indicate whether it binds to a specific protein (output label).

However, there are limited studies that have investigated the role of optimization in computational biology areas, especially in the problem of RNA–protein binding prediction. To bridge this gap, we investigate how random search optimization, grid search, and Bayesian optimizer can contribute to achieve better hyperparameters, automatically fine-tuned for selected datasets. To the best of our knowledge, the role of the CNN optimization method in increasing the accuracy of RBP site prediction has not been evaluated.

This paper provides the following contributions: (1) it presents an empirical evaluation of three gold-standard hyperparameter-tuning optimization methods, random search optimizer, grid search, and Bayesian optimizer, in the context of RNA–protein-binding prediction. We investigate the impact of the hyperparameter-tuning method on improving the model performance. (2) It presents an optimized deep CNN model for RNA–protein binding site prediction.

Our focus in this study is the task of recognizing RNA sequences with RBP binding sites. However, it is worth mentioning other related tasks, such as identifying RNA–protein interactions that detect protein sites binding to RNA [31,32,33,34,35] and the prediction of residue–base contacts between proteins and RNAs [36,37]. The distinction between our task and the other two related tasks lies primarily in the directionality of the interaction being analyzed. Predicting RNA-binding sites on proteins focuses on identifying regions on RNA-binding proteins that are likely to interact with RNA molecules. It involves predicting specific amino acid residues or structural domains on the protein that are involved in binding to RNA. This can help understand protein–RNA interactions, protein function, and potentially aid in drug design or modifying protein behavior. Conversely, predicting protein-binding sites on RNA aims to identify regions on RNA molecules that are likely to interact with specific proteins. It involves predicting RNA sequences or structural motifs that serve as binding sites for particular proteins. Understanding these sites is crucial for deciphering RNA–protein interactions and their roles in various biological processes.

To facilitate navigation, this paper is structured as follows: We begin by outlining the overall research methodology, encompassing the data preprocessing steps and preparation of the random search optimizer, grid search optimizer, and Bayesian optimizer. Next, we delve into the optimized CNN model designed for RPB prediction, followed by a thorough discussion of our empirical findings.

## 2. Materials and Methods

In this paper, an optimized RNA–protein-binding CNN prediction model is proposed using the CLIP-Seq 21 dataset. Three optimization methods are employed, and the empirical results are reported. Optimization approaches have been proven to be efficient means of finding optimal hyperparameters and training choices for deep learning CNN models. In this study, we investigate three very widely used optimization approaches, namely, grid search, random search optimizer, and Bayesian optimizer, on an RNA–protein-binding CNN prediction model. First, the overall model architecture and the empirical approach to modeling the RNA–protein-binding problem is demonstrated. Secondly, we walk through the preprocessing steps to prepare the CLIP-Seq 21 dataset. Next, we present the optimized CNN model with an emphasis on random search, grid search, and Bayesian optimizer to develop the CNN model.

### 2.1. Model Architecture

**Figure 2:**
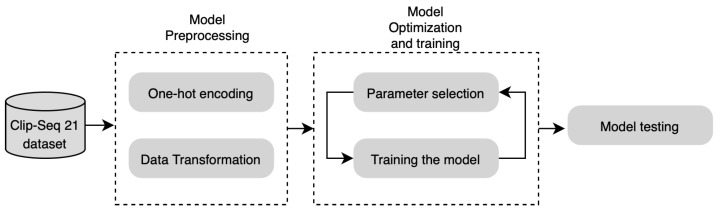
Model architecture.

In this study, we compare the performance of three widely used optimization techniques for hyperparameter tuning of machine learning models: grid search, random search, and Bayesian optimization. We investigate a set of hyperparameters including learning rate, activation function, number of neurons, optimizer, dropout rate, etc. Such hyperparameters can significantly influence the performance of a machine learning model. Our goal is to find the combination of these hyperparameters that results in the best model performance. In this section, we present the modeling approach for predicting RBP binding sites using hyperparameter optimization methods. The modeling process begins with essential preprocessing steps required to prepare the dataset. Initially, we encode the sequence and secondary structure using one-hot encoding. These encoded representations are then input into CNNs to capture abstract motif features. Subsequently, the learned abstract features are utilized in a classification layer to predict RBP binding sites on RNAs. We empirically assess three optimization methods to evaluate the overall model performance and to analyze the impact of random search optimizer, grid search optimizer, and Bayesian optimizer on classification learning and hyperparameter tuning. Our comprehensive evaluation is based on verified RBP binding sites obtained from the large-scale representative CLIP-Seq datasets. The overall architecture of the proposed model is illustrated in Figure 2.

### 2.2. Preprocessing

**Figure 3:**
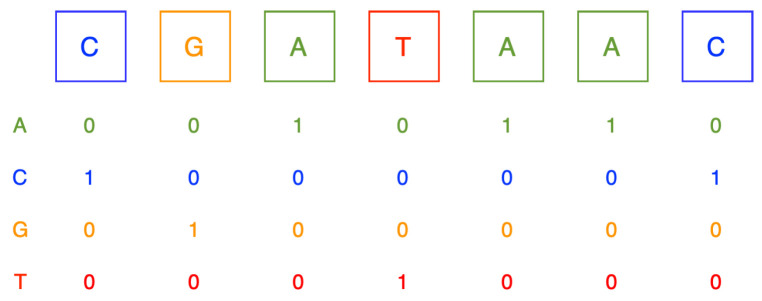
One-hot encoding.

Various algorithms in machine learning exhibit limitations in directly processing label data. Instead, they necessitate the conversion of all input and output variables to numerical formats. In developing an optimized RNA–protein-binding CNN prediction model, we first encode the RNA sequence and secondary structure into one-hot encoding. It is highly important for a machine learning model that categorical data are transformed into one-hot encoding where binary features are created for different categories of the data, i.e., one-hot encoding is a vector representation of a categorical label or feature. This constraint ensures better implementation and modeling of the machine learning problem, as it learns more efficiently with numeric forms of data. An illustration of one-hot encoding is presented in Figure 3.

## 3. Model Optimization

Hyperparameters are external configurations that influence the learning process but are not learned from the data. Hyperparameter tuning is simply searching for the best model architecture from the parameter space to reach the optimal model accuracy. It is considered the most challenging task in developing a machine learning model [38]. Many scientists adopt a trial and error approach to choosing hyperparameters. However, this approach is time consuming, especially for high-dimensional data where complexity expands with each model training iteration. This is true specifically for deep learning models that may not reach local minima [39]. In this section, we investigate three of the most well known hyperparameter-tuning/optimization methods that have proven to be successful with deep learning models; namely, grid search optimizer, random search optimizer, and Bayesian optimizer.

### 3.1. Grid Search

Grid search is a hyperparameter-tuning technique employed in machine learning to systematically explore a predefined set of hyperparameter values for a given model. In a grid search, a discrete set of values for each hyperparameter of interest is specified, creating a multidimensional grid. The algorithm then iteratively trains and evaluates the model with each combination of hyperparameter values within the defined grid. Let Θ represent the set of hyperparameters, where $\theta_{i}$ denotes the *i*-th hyperparameter, and $V_{i}$ represents the set of values considered for $\theta_{i}$. The grid search process can be described as follows: $\mathrm{For}\;\theta_{1}\;\mathrm{in}\;V_{1},\;\mathrm{for}\;\theta_{2}\;\mathrm{in}\;V_{2},\ldots ,\;\mathrm{for}\;\theta_{n}\;\mathrm{in}\;V_{n}$, train and evaluate the model with hyperparameters $\left\{\theta_{1},\theta_{2},\ldots ,\theta_{n}\right\}$. This exhaustive exploration allows one to systematically assess the performance of the model across the entire hyperparameter space, which helps to identify the combination that optimizes the chosen evaluation metric. Grid search is reliable, easy to implement, and has proven to be efficient in low-dimensional spaces [40,41]. Its systematic approach provides a comprehensive understanding of how different hyperparameter values impact model outcomes. However, it can be computationally expensive with an increase in search space dimensionality [42].

### 3.2. Random Search

**Figure 4:**
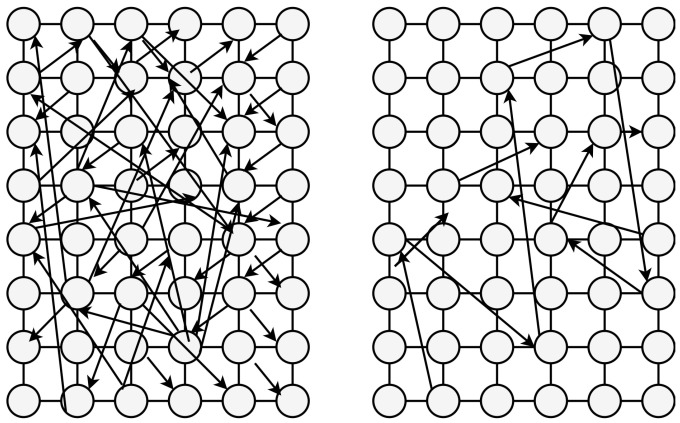
Random optimization vs. Bayesian optimizer.

Random search is a method that involves employing random combinations of hyperparameters to discover the optimal configuration for a constructed model. While akin to grid search, random search has shown to produce superior outcomes in comparison. However, a drawback of random search is its propensity to yield higher computational variance. Given the entirely random selection of parameters, and the absence of a systematic sampling approach, luck plays a role in its effectiveness. A visual representation of the search patterns of random search is illustrated in Figure 4. A walk through the main steps in random search follows:

1. Define a hyperparameter search space;
2. Specify the number of samples;
3. Randomly select a combination of hyperparameters from the predefined search space;
4. Train and evaluate the model;
5. Select best configuration.

As random values are chosen at each instance, there is a substantial probability that the entire action space has been explored due to this randomness, although it can be quite time consuming to cover every possible combination during grid search. This approach is most effective when it is assumed that not all hyperparameters hold equal importance. In this search pattern, random parameter combinations are evaluated in each iteration. The likelihood of discovering the optimal parameters is relatively greater in random search due to its randomized exploration approach, potentially allowing the model to be trained on optimized parameters without encountering aliasing issues. Aliasing occurs when different combinations of hyperparameters result in similar models in terms of performance.

### 3.3. Bayesian Optimization

In contrast to random approaches, Bayesian methods maintain a history of previous evaluation outcomes, which they utilize to construct a probabilistic model linking hyperparameters to the likelihood of achieving a specific score on the objective function. This model is referred to as a “surrogate” for the objective function, denoted as p(y|x). The surrogate function is notably more amenable to optimization compared to the original objective function. Bayesian techniques operate by identifying the next set of hyperparameters to test on the actual objective function, selecting those hyperparameters that exhibit superior performance on the surrogate function. A walk through the steps of Bayesian optimization follows:

1. Build a surrogate probability model of the objective function (often through a Gaussian process (GP));
2. Find the hyperparameters that perform best on the surrogate;
3. Apply these hyperparameters to the true objective function. An acquisition function $x_{next}=argmax_{x}a\left(x\right)$ is used to determine the next point to evaluate the objective function;
4. Update the surrogate model incorporating the new results after the evaluation of the objective function;
5. Repeat steps 2–4 until max iterations or time is reached.

Bayesian optimizer proves useful in optimizing functions that lack differentiability, exhibit discontinuities, and demand significant time for evaluation. The algorithm internally maintains a Gaussian process model to represent the objective function, employing the evaluations of the objective function to refine this model. The fundamental principle underlying Bayesian reasoning is the pursuit of becoming progressively more accurate as more data becomes available. This is achieved through the continual refinement of the surrogate probability model following each evaluation of the objective function. Broadly speaking, Bayesian optimizer methods are efficient due to their judicious selection of the next set of hyperparameters. The core concept is to invest a bit more time in the hyperparameter selection process to minimize the overall calls made to the objective function. A comparison of the parameter searching approach between random optimization and Bayesian optimizer is illustrated in Figure 4.

## 4. Optimized RNA–Protein-Binding CNN Prediction Model

CNNs have recently exhibited impressive performance on non-image data, prompting the consideration of this approach in our experiment. Our data are three-dimensional, comprising instances, window length, and one-hot encoding for ACGT. To make it compatible with 2D CNNs, we transformed it into a four-dimensional format: instances, window length, one-hot encoding for ACGT, and a depth dimension of 1. Numpy was employed for this reshaping process, ensuring compatibility with CNNs. For hyperparameter optimization, we utilized the scikit-learn Python machine learning library. An important element of CNNs is the kernel, which employs shared parameters for each window, significantly reducing computational parameters and training time. CNNs process data batch by batch and window by window within each batch.

After iterating in this manner across the entire image, a feature map, known as a convolved feature map, is formed. To reduce the dimensions of the feature map, and consequently, the number of parameters and computational load, pooling layers are employed. These layers condense the features found within sections of the feature map generated by the preceding convolutional layer. As a result, subsequent operations work on summarized features rather than the precisely located features produced by the convolutional layer. This enhances the model’s robustness to minor shifts in the placement of features within the input image. Various approaches exist for implementing pooling layers, including max pooling, average pooling, and global average pooling.

Max pooling selects the highest-valued element within a defined region of the feature map, producing a new smaller feature map containing the most prominent features from the previous layer. In contrast, average pooling calculates the average value of all elements in the same region, resulting in a feature map with smoothed representations of the original features that captures the general trends of the original features.

In contrast to conventional pooling approaches, global average pooling aims to replace fully connected layers in classic CNNs. It accomplishes this by generating one feature map for each category in the final convolution layer instead of adding additional fully connected layers on top. This not only simplifies the architecture but also fosters a more natural alignment between feature maps and categories. Consequently, each feature map can be viewed as a “category confidence map”, providing interpretable insights into the model’s decision-making process. Furthermore, global average pooling eliminates the need for parameter optimization at this layer, thereby mitigating the risk of overfitting. For these reasons, we selected global average pooling in our proposed model.

**Figure 5:**
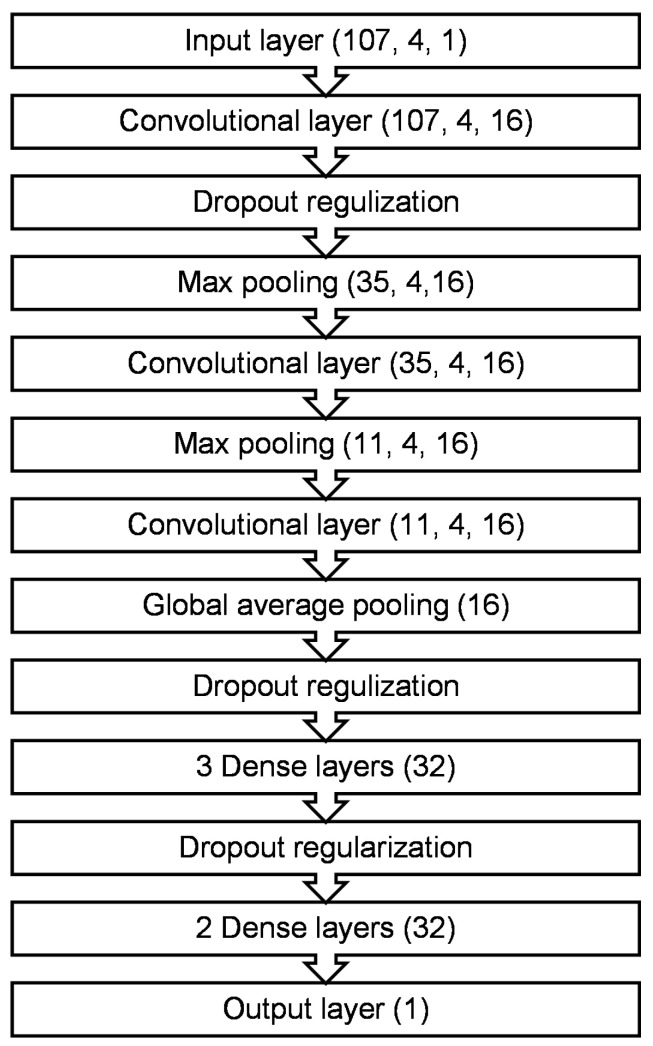
CNN model architecture.

Figure 5 illustrates the proposed CNN architecture. The input is a single RNA sequence, represented as a 3-dimensional array. The first dimension has size 107 (indicating the RNA sequence length), the second dimension uses one-hot encoding to represent the four RNA bases (A, C, G, and T), and the third dimension corresponds to a single instance. The parameters are optimized by the keras_tuner library using three methods: grid search, random search optimizer and Bayesian optimizer. Dropout was used in our architecture to minimize the occurrence of overfitting issues.

## 5. Results

In this section, we present the experimental setup and empirical results of investigating the role of optimization in RBP with three widely used hyperparameter optimization algorithms; namely, grid search, random search, and Bayesian optimization. First, we outline the obtained AUC on 24 datasets using the CLIP-Seq 21 datasets. Then, we investigate further the variation in performance by conducting a comparative study using four datasets. With each dataset, we run the proposed CNN model prior to and after using random search optimizer, grid search optimizer, and Bayesian optimizer. We observe the A receiver operating characteristic curve, or ROC plots to visually analyze and diagnose the classifier improvement spanning different epochs. For each run, we record the performance during testing and training to investigate any overfitting indications. The area under the curve (AUC) is used as a performance metric that measures the classifier’s ability to distinguish classes. A higher AUC score is an indication of better classifier performance.

### 5.1. Experimental Setup

The implemented classifiers were built using open-source machine learning libraries: Keras [43], Tensorflow [44], and supplementary Python libraries. The experiments were run on a multi-core processor and highly computational GPUs (RTX and Tesla) utilizing high-performance AWS EC2 GPU instances.

To validate the performance of the proposed model, we report the testing results of the AUC scores of the proposed CNN model with random search optimizer across 24 experiments on the RBP-24 dataset [45]. For each experiment of the RBP-24 dataset, the dataset is balanced, with 50% positive samples and 50% negative samples. In addition, each experiment is split into 90% for training and 10% for testing. The total number of samples for each experiment varies, with a minimum of 2410 samples for ALKBH5 and a maximum of 238,888 samples for ELAVL1C.

### 5.2. Empirical Results

The empirical results are shown in Table 1 with a mean AUC of 85.30. The proposed model achieved the best AUCs of 94.42%, 93.78%, 93.23%, and 92.68% on the ELAVL1C, ELAVL1B, ELAVL1A, and HNRNPC datasets, respectively.

**Figure 6:**
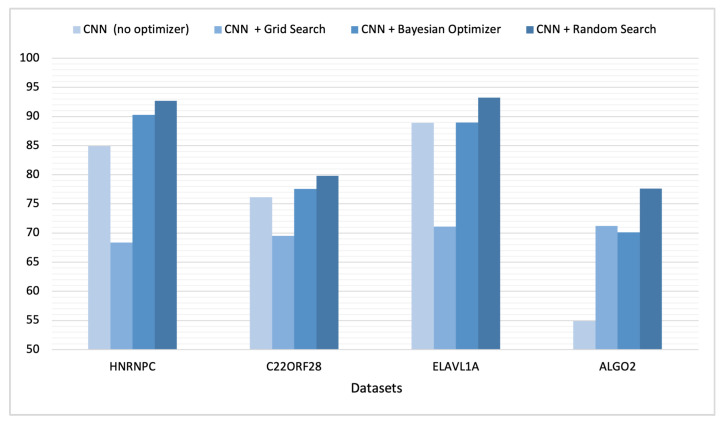
Model performance comparison prior to and after optimization.

Due to the complexity of the datasets and the extended runtime required for executing each experiment with various optimization methods across all datasets, we chose to conduct an empirical study with four randomly selected datasets. The aim was to further explore how optimization affects deep learning models for RNA prediction. These datasets included HNRNPC, C22ORF28, ELAVL1A, and AGO2. We initially created our deep CNN model without using automated optimization and documented the experimental AUC results. Following that, we repeated the experiment for the aforementioned datasets, employing the three optimization methods reported in Table 2. Our empirical results indicate a measurable increase in the AUC score after employing random search optimizer and Bayesian optimizer. Table 2 and Figure 6 outline a comparison of the deep learning models’ performance prior to and after employing optimization.

**Figure 7:**
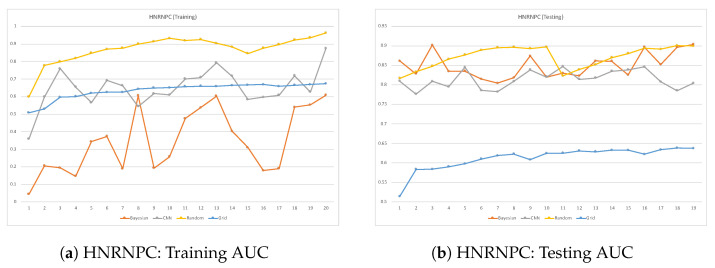
Training vs. testing loss for HNRNPC dataset.

**Figure 8:**
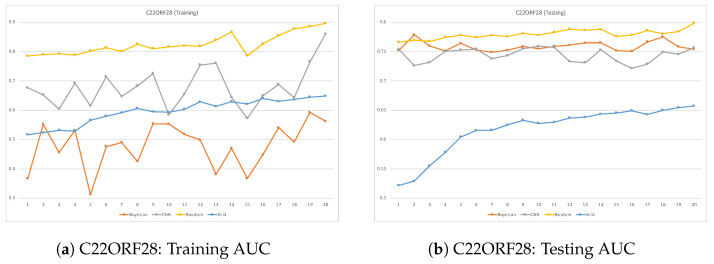
Training vs. Testing loss for C22ORF28 dataset.

**Figure 9:**
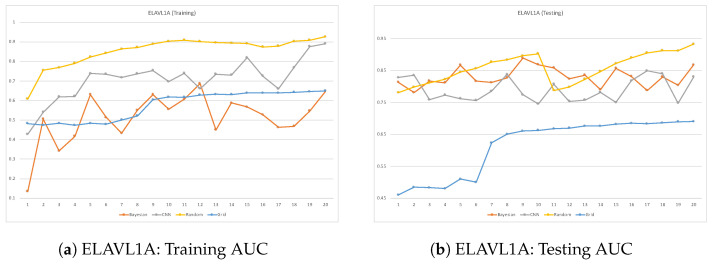
Training vs. testing loss for ELAVL1A dataset.

**Figure 10:**
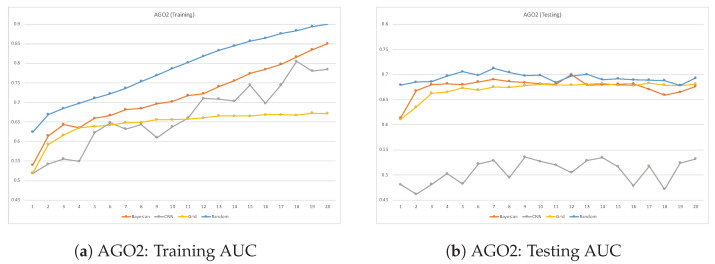
Training vs. testing AUC for AGO2 dataset.

ROC plots are used to compare the classifier performance during testing vs. training when running each dataset. Such a comparison can be accomplished by comparing the classifier’s AUC performance over many epochs during training and testing. When a classifier reaches a high AUC during the training phase but significantly drops in the testing phase, it is considered a sign of overfitting. This issue takes place when deep learning models learn well during training but fail to generalize in the testing phase. Figure 7, Figure 8, Figure 9 and Figure 10 are the ROC plots demonstrating the performance on the four RPB datasets, HNRNPC, C22ORF28, ELAVL1A, and AGO2, during training and testing. In each plot, the performance of the CNN model without hyperparameter optimization is illustrated with a blue line running over multiple epochs. The orange and the blue lines illustrate the prediction performance of the CNN model with Bayesian optimizer and random search optimizer, respectively. The yellow line illustrates grid search optimizer. Grid search optimizer, however, outperformed the CNN model with no optimizer on the testing set on HNRNPC and AGO2 and fell short on the ELAVL1A and C22ORF28 datasets. Mostly, applying hyperparameter optimization on the CNN classification model demonstrated an improved mapping of hyperparameter values, hence improving the prediction capability of the CNN model.

In all datasets, it was prominently apparent that random search optimizer achieved a better performance compared to Bayesian optimizer and grid search. Our analysis showed that the plain CNN model’s performance during training outperformed the one with Bayesian optimizer. However, this observation does not hold true during model testing, where it shows clear signs of overfitting problems. However, random search optimizer and Bayesian optimizer demonstrate a more stable trade-off between training and testing. All in all, the proposed CNN model with random search optimizer showed the best learning capability among the trained models.

## 6. Conclusions

In this paper, we investigated the impact of three optimization methods on RNA–protein-binding prediction, which is an important problem investigated in the field of bioinformatics and chemoinformatics related to the effect of RNA and protein binding on gene expression. We empirically tested multiple datasets and showed that hyperparameter optimization techniques improved the model performance and had a positive impact on model learning. Our approach also minimizes the time researchers need to spend trying to tune machine learning problems for different tasks. First, we introduced an optimized deep CNN model for RNA–protein binding site prediction. Then, we delivered empirical results of the optimization techniques, shedding light on their pivotal role in refining the performance of deep learning prediction models on the CLIP-Seq 21 dataset.

Moreover, an empirical comparative analysis was presented, demonstrating the effectiveness of random search optimizer, grid search, and Bayesian optimizer within the context of the CNN model for RNA–protein binding site prediction. This comparative study not only highlights the advantages of optimization but also provides valuable insights into the nuanced interplay between optimization strategies and model performance.

This research advances our understanding of RNA–protein binding site prediction by proving the impact of optimization techniques. It serves as a valuable resource for researchers and practitioners in the field, paving the way for more accurate and efficient predictive models in RNA–protein interaction studies. The deep learning models presented in this study address the task of recognizing RNA sequences with RBP binding sites. Further downstream analysis involves using alignment algorithms to locate and display binding motifs. Our models could be integrated into a comprehensive framework, to be investigated in future work.

## Figures and Tables

**Table 1:** Empirical results of the proposed CNN model with random search optimizer.

#	RBP	Optimized CNN Model
1	ALKBH5	66.8
2	C17ORF85	75.2
3	C22ORF28	79.8
4	CAPRIN1	76.8
5	Ago2	77.6
6	ELAVL1H	91.26
7	SFRS1	88.42
8	HNRNPC	92.68
9	TDP43	90.25
10	TIA1	84.89
11	TIAL1	84.83
12	Ago1-4	85.56
13	ELAVL1B	93.78
14	ELAVL1A	93.23
15	EWSR1	88.4
16	FUS	93.2
17	ELAVL1C	94.42
18	IGF2BP1-3	78.24
19	MOV10	82.83
20	PUM2	88.32
21	QKI	83.82
22	TAF15	88.40
23	PTB	89.76
24	ZC3H7B	78.92
	**Mean**	**85.30**

**Table 2:** CNN model’s AUC results with different optimizers.

Dataset	CNN Model (No Optimizer)	CNN+ Grid Search	CNN+ Bayesian Optimizer	CNN+ Random Optimization
HNRNPC	84.9	68.4	90.28	**92.68**
C22ORF28	76.16	69.5	77.57	**79.8**
ELAVL1A	88.931	71.1	88.97	**93.23**
ALGO2	54.92	71.2	70.14	**77.62**

## Data Availability

The dataset used in this study is publicly available at http://www.bioinf.uni-freiburg.de/Software/GraphProt/GraphProt_CLIP_sequences.tar.bz2 (accessed on 1 September 2023).
